# Atypical Calcitriol-Mediated Hypercalcemia in Late Life: A Hypothesis-Generating Case

**DOI:** 10.7759/cureus.92022

**Published:** 2025-09-10

**Authors:** Hsien Yi Yang, Cyrus Nensey

**Affiliations:** 1 Hospital Medicine, Northridge Hospital Medical Center, Los Angeles, USA

**Keywords:** acute hypercalcemia, altered mental state, fgf-23, hospice and palliative care, klotho

## Abstract

Calcitriol-mediated hypercalcemia is most commonly associated with granulomatous disease or lymphoma and rarely occurs without an identifiable cause. We present the case of a 96-year-old woman who developed severe symptomatic hypercalcemia with elevated 1,25-dihydroxyvitamin D (calcitriol), low 25-hydroxyvitamin D (calcidiol), suppressed parathyroid hormone, and no evidence of malignancy, granulomatous disease, endocrine dysfunction, or vitamin D intoxication. She improved with intravenous fluids, calcitonin, and corticosteroids. We hypothesize that age-related impairment of the fibroblast growth factor 23-Klotho regulatory axis contributes to the dysregulation of calcitriol synthesis. This case highlights diagnostic challenges in elderly patients with hypercalcemia and raises the possibility of age-related mineral metabolism dysregulation as an underrecognized contributor to idiopathic calcitriol-induced hypercalcemia.

## Introduction

Hypercalcemia is classified as parathyroid hormone (PTH)-dependent or PTH-independent. PTH-dependent hypercalcemia is most often due to primary hyperparathyroidism [1]. PTH-independent hypercalcemia is frequently caused by malignancy through osteolytic metastases, parathyroid hormone-related peptide (PTHrP) secretion, or ectopic calcitriol production [1]. Other mechanisms include excess vitamin D intake, granulomatous disease, endocrine disorders, and drug-induced causes [1]. Calcitriol-mediated hypercalcemia is uncommon. It typically occurs in granulomatous inflammation or lymphoma due to unregulated extrarenal 1α-hydroxylase activity [2]. Glucocorticoids are effective because they suppress extrarenal calcitriol production [2]. Idiopathic (without an identifiable cause) calcitriol-induced hypercalcemia is rare. It accounts for approximately 3% of calcitriol-mediated cases, and its pathogenesis remains unclear [2].

The report describes the case of an elderly patient with idiopathic calcitriol-induced hypercalcemia. We propose a role for age-related impairment of the fibroblast growth factor 23 (FGF23)-Klotho axis in the dysregulation of vitamin D metabolism.

## Case presentation

A 96-year-old woman with hypertension, atrial fibrillation, and prior transcatheter aortic valve replacement presented to our hospital after a mechanical fall and generalized weakness. At baseline, she was alert and oriented to person, place, and time, despite intermittent forgetfulness. She was ambulatory with a front-wheel walker and able to eat independently with a spoon. She lived at home with assistance. The family denied recent supplementation with vitamin D or vitamin A. Home medications included celecoxib, topical diclofenac, escitalopram, famotidine, furosemide, metoprolol, pregabalin, rosuvastatin, and pancrelipase.

On arrival, she was difficult to arouse and disoriented. Laboratory evaluation revealed hypercalcemia, suppressed PTH, elevated calcitriol, low calcidiol, normal phosphorus and alkaline phosphatase levels, mildly elevated angiotensin-converting enzyme (ACE), mildly elevated thyroid-stimulating hormone (TSH), and mildly elevated free T4. She was also noted to have mildly elevated cortisol as well as acute kidney injury (Table 1).

**Table 1:** Pertinent laboratory values related to calcium and endocrine metabolism on admission. PTHrP, parathyroid hormone-related protein; TSH, thyroid-stimulating hormone

Investigation	Result	Normal Range
Calcium and vitamin D metabolism		
1,25(OH)2D (pg/mL)	148.0	19.9-79.3
25(OH)D (ng/mL)	26	30-100
Intact parathyroid hormone (pg/mL)	5	15-65
Calcium (mg/dL)	13.6	8.5-10.0
Corrected calcium (mg/dL)	14.88	8.50-10.00
Ionized calcium (mmol/L)	1.80	1.16-1.32
Phosphorus (mg/dL)	3.7	2.4-4.4
PTHrP (pmol/L)	2.5	0-3.4
Alkaline phosphatase (U/L)	62	30-100
Renal function and metabolic status		
Lactic acid (mmol/L)	5.5	0.5-2.2
Creatinine (mg/dL)	1.13	0.40-1.00
Endocrine function		
Angiotensin-converting enzyme (U/L)	91	16-85
Cortisol (mcg/dL)	26.3 (3:34 PM)	3.0-16.0 (4 PM)
Free T4 (ng/dL)	1.45	0.60-1.20
TSH (mcIU/mL)	4.45	0.40-4.00

**Figure 1:**
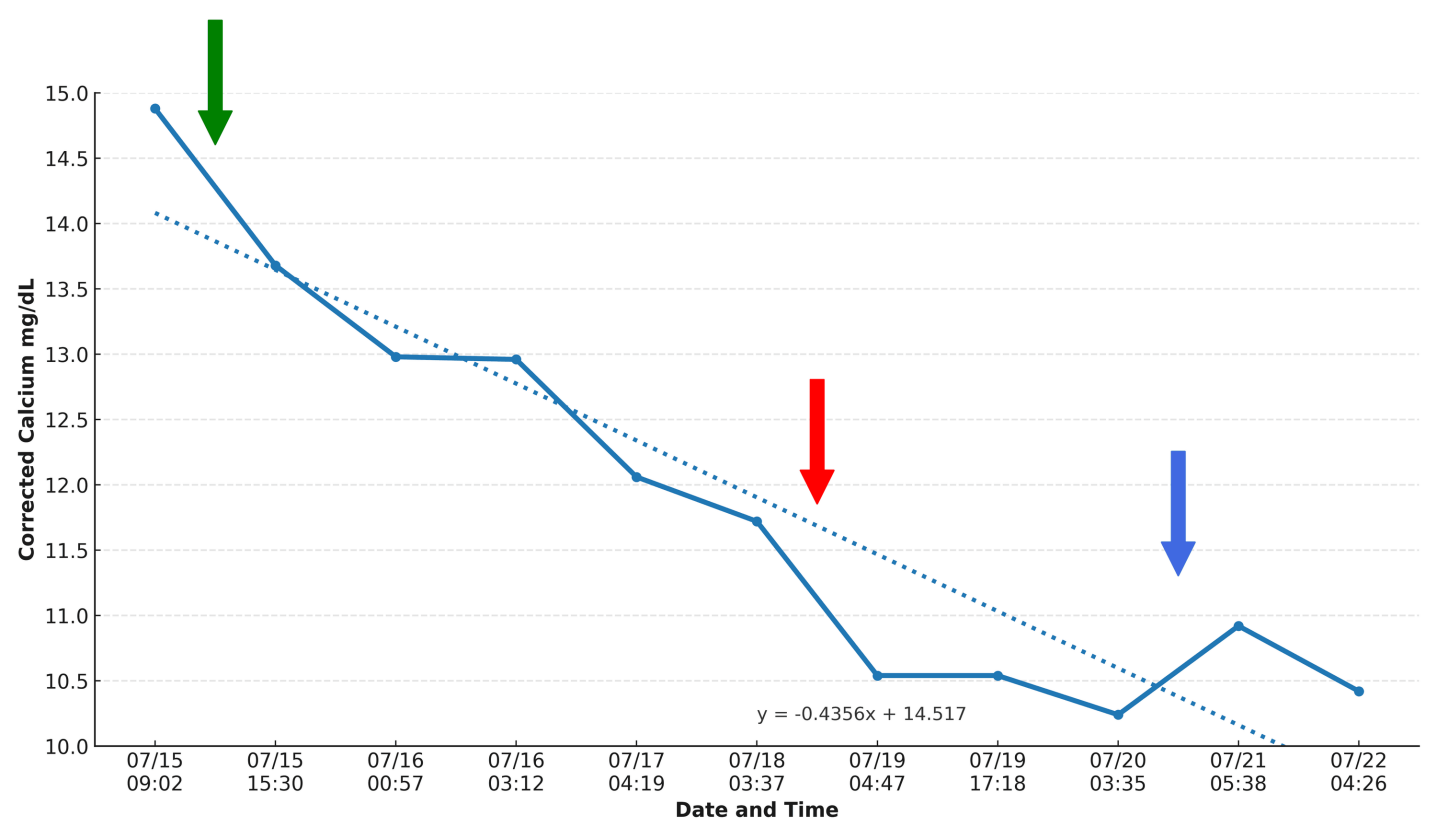
Calcium levels throughout the hospital course. The green arrow indicates IV fluid started, the red arrow indicates that one dose of calcitonin was given, and the blue arrow indicates that steroid therapy started. Corrected calcium (mg/dL) = measured serum calcium (mg/dL) + [0.8 × (4.0 − serum albumin (g/dL))]

She was treated with intravenous (IV) fluids, intermittent IV furosemide (discontinued due to rising creatinine), calcitonin, and corticosteroids. Initially, she was started on methylprednisolone and later switched to prednisone. Bisphosphonate therapy was considered but deferred given renal impairment [3]. Overall, the serum corrected calcium decreased from 14.88 mg/dL to 10.2 mg/dL over seven days, accompanied by improvement in her mental status (Figure 1).

**Figure 2:**
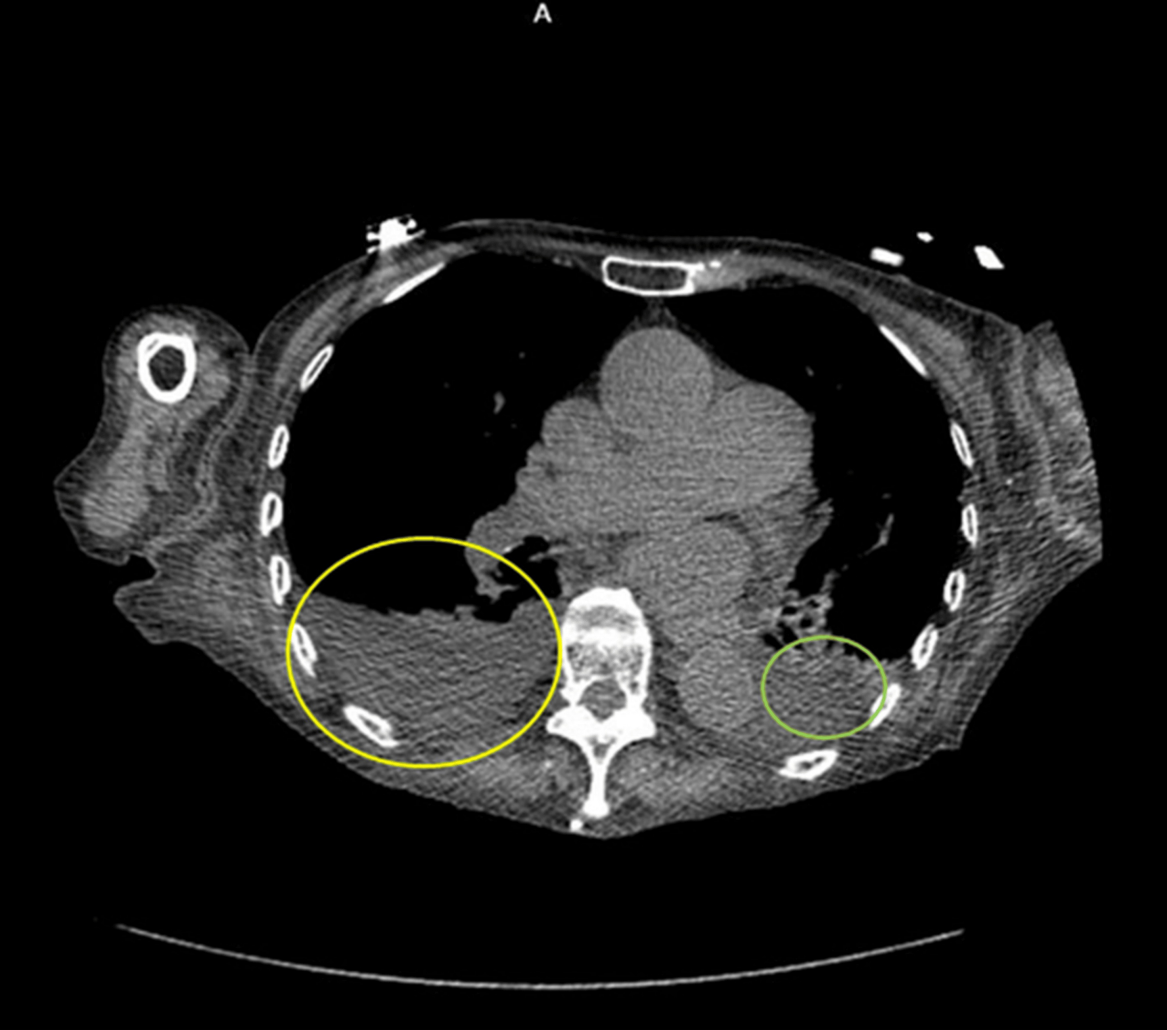
Axial chest CT without contrast. Small-to-moderate right (yellow) and small left (green) pleural effusions are seen, with no mediastinal calcification or lymphadenopathy.

**Figure 3:**
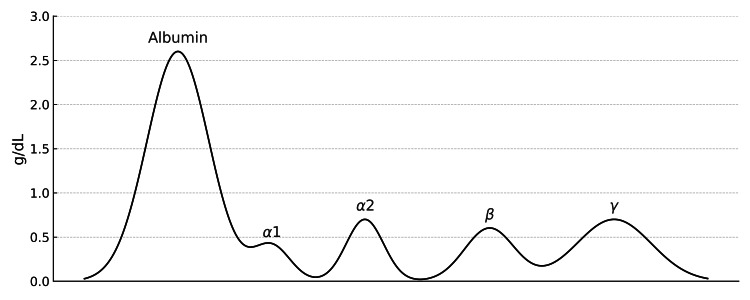
Serum protein electrophoresis (SPEP) The overall pattern demonstrates a low albumin-to-globulin ratio with no dominant monoclonal spike.

**Figure 4:**
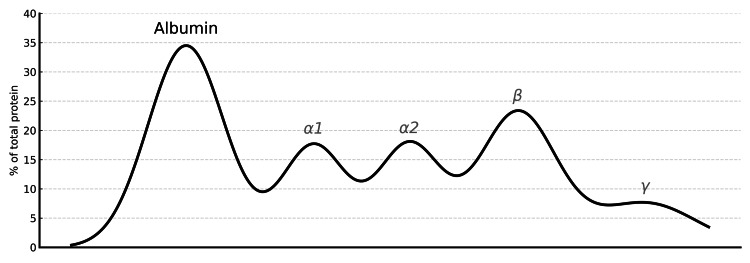
Urine protein electrophoresis (UPEP) No monoclonal spike is present, and albumin makes up most of the urinary protein.

**Figure 5:**
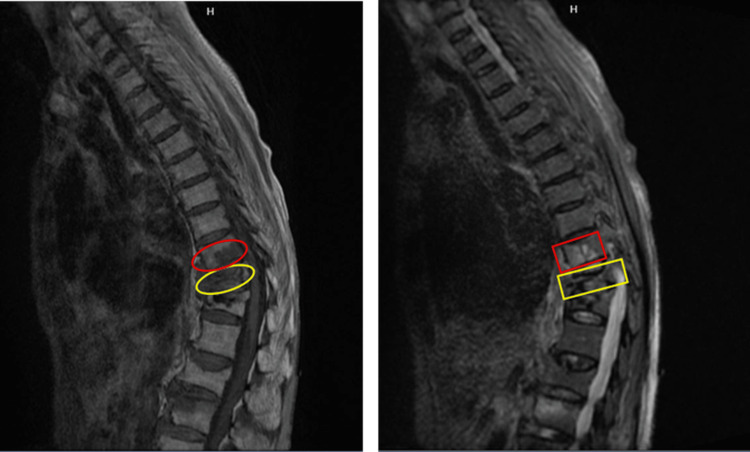
MRI of the thoracic spine without contrast: T1-weighted (left) and STIR (right) images. Red marks indicate T10 changes consistent with a compression fracture; yellow marks highlight T11 changes with a severe vertebral body fracture. STIR, short tau inversion recovery

**Figure 6:**
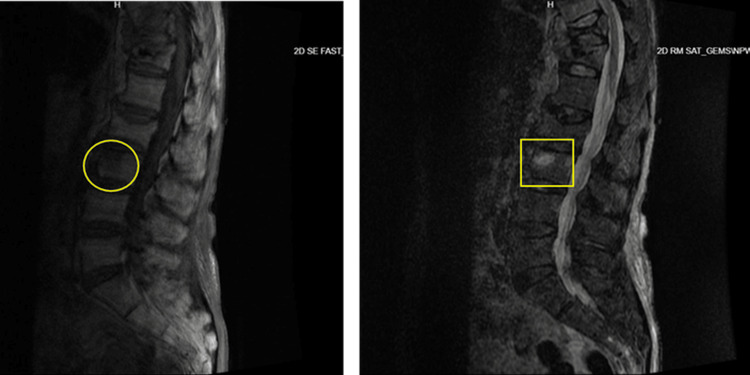
MRI of the lumbar spine without contrast: T1-weighted (left) and STIR (right) images. Yellow marks indicate a compression fracture at L3. STIR, short tau inversion recovery

Chest computed tomography (CT) revealed moderate right and small left pleural effusions without cavitation, lymphadenopathy, or mass (Figure 2). The fungal study was negative (Table 2). Tuberculosis testing was consistent with known latent tuberculosis (Table 2). Serum and urine protein electrophoresis (SPEP and UPEP) were negative for monoclonal proteins (Figures 3, 4). Magnetic resonance imaging (MRI) of the thoracic and lumbar spine revealed acute-to-subacute compression fractures at T10 and L3 without evidence of marrow infiltration, findings consistent with her trauma history (Figures 5, 6). Given poor oral intake, nasogastric (NG) tube feeds were initiated. Despite partial recovery, her functional status remained impaired. After goals-of-care discussion, the family elected for comfort-focused care, and she was discharged to hospice. At discharge, prednisone therapy was maintained at 40 mg daily, with a planned taper of 10 mg every three days. Further diagnostic studies were not pursued due to the hospice decision.

**Table 2:** Pertinent infectious disease laboratory values on admission.

Investigation	Result	Normal Range
Endemic mycoses		
Histoplasma yeast	<1:8	<1:8
Histoplasma mycelia	<1:8	<1:8
Coccidioides antibody	<1:2	<1:2
Tuberculosis		
Quantiferon NIL (IU/mL)	0.315	Not applicable
Quantiferon mitogen-NIL (IU/mL)	>10.000	Not applicable
Quantiferon TB1 antigen-NIL (IU/mL)	0.707	0-0.340
Quantiferon TB2 antigen-NIL (IU/mL)	0.555	0-0.340

## Discussion

This patient’s presentation with severe symptomatic hypercalcemia, elevated calcitriol, low calcidiol, and suppressed PTH is consistent with PTH-independent, calcitriol-mediated hypercalcemia [2]. Several major categories were evaluated in the differential diagnosis.

Granulomatous disease such as sarcoidosis, tuberculosis, and fungal infection was considered. Serologic testing was negative for endemic mycoses (Table 2). The chest CT revealed no consolidation, cavitation, lymphadenopathy, or mediastinal mass, making sarcoidosis and active tuberculosis infection unlikely (Tables 1, 2, Figure 2). Malignancy was also evaluated. Lymphoma and multiple myeloma were excluded by negative serum and urine protein electrophoresis results and the absence of lytic lesions (Figures 3, 6). Medication-induced hypercalcemia was ruled out, given the absence of recent vitamin D, vitamin A, thiazide, or calcium supplement use. Familial hypocalciuric hypercalcemia was unlikely given her advanced age and acute presentation [4].

Endocrine and metabolic disorders were also assessed. Thyroid studies supported euthyroid sick syndrome, and cortisol elevation reflected a physiologic stress response (Table 1). In addition, normal phosphorus and alkaline phosphatase levels argued against granulomatous disease, metabolic bone disorders, or high bone turnover states such as hyperparathyroidism or metastatic disease (Table 1).

Our case shares similarities with a previously reported 51-year-old male with recurrent nephrolithiasis and acute kidney injury [5]. Both patients had elevated calcitriol, suppressed PTH, low calcidiol, and negative evaluations for malignancy or granulomatous disease, and responded to corticosteroids [5]. In contrast, our patient was of advanced age, with an acute symptomatic presentation and functional decline, complicated by volume overload and renal impairment due to structural heart disease.

In the absence of common causes, we considered age-related mechanisms affecting vitamin D regulation. Klotho is a transmembrane protein that serves as the co-receptor for FGF23 in phosphate and vitamin D metabolism [6]. Both Klotho and FGF23 suppress renal and extrarenal 1α-hydroxylase activity, thereby limiting calcitriol synthesis [7,8]. Human studies show that serum Klotho levels decline markedly after age 55 compared with young adults [8]. Murine models demonstrate that deficiency of either Klotho or FGF23 results in unchecked calcitriol production, hypercalcemia, and phosphate imbalance [7,9]. A rare human case of tumoral calcinosis was linked to a homozygous missense mutation in the KLOTHO gene [10]. This supports that impaired Klotho function disrupts FGF23-mediated phosphate and vitamin D homeostasis [10]. We hypothesize that aging impairs the fibroblast growth factor 23-Klotho axis, leading to dysregulated calcitriol synthesis.

Corticosteroids were initiated after IV fluids, diuresis, and calcitonin, at which point calcium had already begun to decline. Following steroid initiation, calcium levels stabilized without rebound. However, the extent of steroid-specific effect can only be confirmed in the outpatient setting. Considering her age and goals of care, further work-up may not have changed the outcome. However, such evaluation may be worthwhile in future cases. Positron emission tomography-computed tomography (PET-CT) could have helped detect occult malignancy or metabolically active granulomatous disease not seen on conventional imaging. Nevertheless, PET-CT and follow-up calcitriol measurements after steroid initiation were not performed. This case underscores the delicate balance between advancing scientific understanding and honoring patient dignity, reminding us that compassionate care remains central to clinical decision-making.

## Conclusions

Idiopathic calcitriol-induced hypercalcemia is rare and challenging to diagnose, particularly in older adults, and requires exclusion of malignancy and granulomatous disease in PTH-independent presentations. Transition to hospice limited further evaluation, underscoring the importance of balancing ethical considerations with the pursuit of diagnostic certainty. Age-related dysregulation of the FGF23-Klotho axis may contribute, and while early recognition is important, prognosis depends largely on comorbidities and functional reserve.
